# Introduction: New Perspectives in Hereditary Angioedema (HAE): Molecular Mechanisms & Therapeutic Choices: *A CME Symposium Presented at the 2009 World Allergy Congress, Buenos Aires, Argentina, December 9, 2009*

**DOI:** 10.1097/1939-4551-3-S3-S24

**Published:** 2010-09-15

**Authors:** Bruce L Zuraw, Michael M Frank, Marc A Riedl

**Affiliations:** 1 Duke University Medical Center, Durham, North Carolina; 2 Clinical Immunology and Allergy, UCLA-David Geffen School of Medicine, Los Angeles, CA; 3 Department of Medicine, University of California San Diego and San Diego Veteran's Affairs Medical Center, La Jolla, CA

## 

Hereditary angioedema (HAE), an autosomal dominant disorder that occurs in approximately 1 in 50,000 people, is characterized by episodes of swelling that typically affect the extremities, bowels, face, or genitals. This supplement contains 3 articles that address the molecular mechanisms that underlie HAE symptoms and treatment options that are currently available and in development.

There are three types of HAE; Types I and II are the most common. Patients with HAE lack functional C1 inhibitor (C1INH), resulting from mutations that prevent its secretion (type I) or result in the secretion of a nonfunctional protein (type II). C1INH inactivates a number of proteases in the complement and contact systems. In his article on the pathophysiology of HAE, Zuraw delineates how the lack of C1INH dysregulates the contact system in patients with HAE, leading to the overproduction of bradykinin and a subsequent increase in vascular permeability.

Historically few agents have been available for prophylaxis or treatment of HAE, particularly in the United States. Those which have been available, such as the androgens for prophylaxis, are associated with side effects that limit their use in some populations. However, several new treatment options have recently become available or are in the pipeline.

Frank describes the development of C1INH replacement therapy for use both in preventing HAE and in treating acute attacks. Two plasma-derived preparations were recently approved for use in the United States: Cinryze (ViroPharma) for HAE prophylaxis and Berinert (CSL Behring) for treatment of acute attacks. A recombinant protein, Rhucin (Pharming), is currently in phase III trials.

From the ^1^Duke University Medical Center, Durham, North Carolina; ^2^Clinical Immunology and Allergy, UCLA-David Geffen School of Medicine, Los Angeles, CA; and ^3^Department of Medicine, University of California San Diego and San Diego Veteran's Affairs Medical Center, La Jolla, CA.

This CME satellite symposium was kindly supported by an educational grant from ViroPharma Incorporated, and sponsored by the World Allergy Congress 2009 and Robert Michael Educational Institute LLC. In addition, the authors and editors would like to thank Naomi Ruff, PhD, for her editorial contribution.

Increased understanding of how a deficit of C1INH leads to angioedema has provided additional therapeutic targets. Riedl describes the development of 2 new drugs targeting elements in the contact system pathway: ecallantide (Kalbitor, Dyax) and icatibant (Firazyr, Shire). Ecallantide specifically inhibits kallikrein, the enzyme that produces bradykinin from high-molecular-weight kininogen; it has been approved in the United States (but not Europe) for the treatment of acute attacks. Icatibant is a specific antagonist of the bradykinin B2 receptor; blocking this receptor prevents bradykinin from acting on vascular endothelial cells to increase their permeability. Icatibant has been approved for use in Europe, and additional clinical trials are underway in the United States.

Because the frequency and severity of HAE symptoms vary widely, additional work will be needed to optimize the care of each patient. However, recent advances in understanding the pathophysiology of HAE and newly available therapies to treat or prevent attacks will provide important and potentially life-saving options for patients with this often disabling condition. 

## Contents

Introduction

Bruce L. Zuraw, Michael M. Frank and Marc A. Riedl

The Pathophysiology of Hereditary Angioedema

Bruce L. Zuraw

Hereditary Angioedema: Recombinant and Purified Human C1 Inhibitors

Michael M. Frank

Review of Hereditary Angioedema Treatments: Kallikrein Inhibitors and Bradykinn Type 2 Receptor Antagonists

Marc A. Riedl

Summary and Conclusions

Bruce L. Zuraw

